# Visuo-motor lateralization in *Apis mellifera*: flight speed differences in foraging choices

**DOI:** 10.1038/s41598-023-51141-w

**Published:** 2024-01-05

**Authors:** Davide Liga, Gionata Stancher, Elisa Frasnelli

**Affiliations:** 1 University of Trento, CIMeC, ING, 38068 Rovereto, TN Italy; 2 Rovereto Civic Museum Foundation, 38068 Rovereto, TN Italy

**Keywords:** Decision, Navigation, Animal behaviour, Entomology, Perception

## Abstract

Evidence of lateralization has been provided in *Apis mellifera* in olfactory learning and social interactions, but not much is known about how it influences visuo-motor tasks. This study investigates visuo-motor biases in free-flying honeybees by analysing left/right choices related to foraging in a Y-maze. Individual bees were trained to associate a visual stimulus (a blue or yellow target) with a reward/punishment: the *Blue* + group was reinforced for the blue and punished for the yellow, and vice versa for the *Yellow* + group. In unrewarded tests, we assessed for each bee the directional choice for one of the two identical targets (12 trials with blue targets and 12 with yellow targets) placed in the left and right arms of the maze as well as the flight times to reach the target chosen. The results did not reveal a significant directional preference at the population level, but only at the individual level, with some individuals presenting a strong bias for choosing the right or left stimulus. However, the data revealed an interesting new factor: the influence of both direction and colour on flight times. Overall, bees took less time to choose the stimulus in the left arm. Furthermore, the yellow target, when previously associated with a punishment, was reached on average faster than the punished blue target, with a higher number of no-choices for punished blue targets than for punished yellow targets. This opens new perspectives not only on the study of lateralization in *Apis mellifera*, but also on the bees’ chromatic preferences.

## Introduction

The concept of lateralization, which refers to the distinct functional specialisation of the left and right hemispheres of the brain, is a characteristic found in both simple and more complex nervous systems. In fact, there is extensive evidence of behavioural and brain asymmetry in both vertebrates^1^ and invertebrates^2,3^. These asymmetries manifest in various ways, including limb use preferences, directional biases, as well as differences in sensory and cognitive abilities. Moreover, they can occur at a population level, where the majority of individuals in a population exhibit the same directional bias (either right or left), or at an individual level, where a similar number of individuals within the population displays lateralization in each direction. Population-level lateralization has been proposed to result from social pressures encountered during evolution^3–5^ and may be linked to behaviours that necessitate interaction and possibly coordination among individuals^6,7^. This idea finds support in studies on insects^1,3,8^ emphasising the importance of investigating lateralization even in simpler nervous systems. An illustrative example of this phenomenon can be observed in the fruit fly, *Drosophila melanogaster*, which displays individual-level motor biases both in Y-maze navigation and circling behaviour^9^. Interestingly, these motor biases are not associated with any observable morphological traits, such as gut twisting laterality, leg length asymmetry, or wing-folding preferences, nor are they linked to specific genetic strains, but, instead, these biases appear to originate centrally, as their strength increases when neurons in the central complex of the brain are silenced^9^. In the species of ants, there are also instances of motor biases. For instance, 12 species of *Lasius* ants tend to keep the right side of their foraging "streets," while one species prefers the left^10^. Another ant species, *Formica pratensis*, which does not rely on trail pheromones, demonstrates a population-level tendency to keep the left to avoid encounters with other workers when returning to the nest with food, but not when leaving the nest to search for food^11^. Among ants, *Temnothorax albipennis* displays a population-level inclination to explore unknown nest sites in a leftward direction^12^.

Similar patterns are observed in honeybees (*Apis mellifera*). They exhibit a population-level motor bias when exploring unfamiliar open cavities, predominantly favouring the right direction (unlike ants, which favour the left^12^), with individuals strongly biased to the right making quicker decisions^13^. Furthermore, individual honeybee workers, when presented with a pair of identical apertures to fly through, display individual-level preferences for either the right or left aperture, with some individuals displaying no preference^14^. Some species of bumblebees, such as *Bombus lapidarius, Bombus lucorum,* and *Bombus pascuorum*, exhibit a preference for circling in either a clockwise or anti-clockwise direction when they visit florets arranged around an upright inflorescence; in contrast, buff-tailed bumblebees (*Bombus terrestris*) do not show such a directional preference^15^. Free-flying *Bombus terrestris* bumblebees trained in a T-maze to associate a specific colour with a sugar reward, when presented with the trained colour at the end of both arms of the maze without a reward, they tend to prefer the right arm^16^. This suggests a visuomotor bias towards this direction in this species.

The behaviour of the large white butterfly (*Pieris brassicae*) displays variations in flight direction (within 360° gauze), both within individuals and between individuals: highly mobile and long-winged individuals tend to fly in similar directions (i.e., within a certain range of degrees—for details see^17^), while less mobile and short-winged individuals exhibit divergent flight directions^17^. Similarly, melon flies (*Zeugodacus cucurbitae*) exhibit individual-level biases in flight direction, with 83% of individuals showing a preference for continuing in a "forward" direction. However, when wind effects were analysed, it became apparent that wind direction was not the sole factor influencing these motor biases, as the mean flight directions matched the wind direction in only 36% of the flies^18^. Studies on visual lateralization have shown interesting results. For instance, octopuses exhibit lateralization in how they use their eyes and arms^19^, while bumblebees exhibit lateralization in visual-motor tasks resembling their foraging behaviour. This suggests a potential specialization of the right side of the nervous system for routine tasks^16^. Honeybees trained to visual tasks using the Proboscis Extension Reflex (PER) exhibit improved learning of coloured stimuli when observed with their right eye rather than their left one^20^. They also display greater precision and efficiency in associating odours with sugary rewards when using their right antenna as opposed to their left one^21^.

In this context, it is noteworthy that bees, including *Apis mellifera, Trigona carbonaria, Trigona hockingsi* and *Austroplebeia australis*, rely on their right antenna for recalling short-term olfactory memories (one hour after training), but the retrieval of long-term memories (at 6 and 23 h after training) primarily engages the use of the left antenna^22–24^. These species also exhibit a preference for their right antenna when interacting with conspecifics^1^ and display variations in their ability to learn olfactory stimuli between the left and right olfactory pathways^21,24–26^. The asymmetries observed in the olfactory realm also appear to impact motor biases in *Apis mellifera*. We know that honeybees exhibit individual biases when choosing between two identically sized openings within a tunnel: some bees exhibit a preference for the right opening, while others favour the left (26.5% for the right aperture, 24.5% for the left aperture, and 49% exhibit no bias^14^). This bias is believed to enhance efficiency within the hive, which is riddled with tunnels and hollows^27^, as well as increases the speed and safety of a group of bees navigating through dense foliage in search of food or during swarming^14^. Furthermore, *Apis mellifera ligustica*, as a population, demonstrates a notable tendency to turn right when exploring unfamiliar cavities^13^. This rightward bias aligns with the antennal physiology of *Apis mellifera*, as the right antenna is preferentially utilized in short-term memory, pheromone detection and social interactions, likely due to its higher density of olfactory sensilla^1,24,25^. Consequently, when bees investigate an open cavity, which may hold both the risk of predation and the potential for social interactions, a right turn is favoured over a left turn^14,27^. This choice enables workers to gather information about their surroundings with greater accuracy^13^. The existing literature seems overall to indicate that honeybees exhibit either individual-level or population-level biases depending on the context with some evidence in favour of a right bias in tasks involving olfactory and visual learning or exploration of new environments. Interestingly, these studies also suggest a possible similarity in lateralization pattern between vertebrates and invertebrates in relation to the preference to approach stimuli and to inspect situations on the right side, and of avoidance on the left side^26,28^.

This study aims at investigating the possible complementary specialization of the right and left sides of the brain in processing visual stimuli eliciting an approach and a withdrawal reaction in a foraging context in *Apis mellifera*. Individual honeybees were trained to associate one colour (blue or yellow) with a sugar reward and another colour (yellow or blue) with a quinine punishment (in balanced groups to control for a possible colour effect). Each bee was then tested in a Y-maze where the same colour (either the previously rewarded one or the punished one) was presented unrewarded in both the right and left arms to establish visuo-motor biases in preferentially choosing one arm over the other. According to the existing literature on different vertebrate and invertebrate species (for a review see^29^) and, in particular, according to a recent study of honeybees showing that they avoid olfactory cues presented on the left side and approach them when presented on the right side^26^, we expected the right arm to be chosen more often when the rewarded colour was presented on the right side and the punished colour to be avoided more often when it was presented on the left side. If so, this might cause a preference to turn into the right arm of the maze.

## Results

When all the choices were analysed regardless of the colour presented and whether it was associated with a reward or a punishment, results revealed a total of 235 left picks, 225 right picks and 44 NAs (i.e., no choice within the time allowed). As regards the NAs, in the *Blue* + group (i.e., rewarded for the blue colour and punished for the yellow) there were 7 NAs all for the punished colour (yellow), while in the *Yellow* + group (i.e., rewarded for the yellow and punished for the blue) there were 37 NAs all related to the punished colour (blue).

Regarding the choice of direction (i.e., right *vs*. left), the data analysis did not show any effect of colour (*LRT, χ*^*2*^_*1*_ = *0.67, p* = *0.413*), nor of reward/punishment (*LRT, χ*^*2*^_*1*_ = *1.46, p* = *0.227*), or of their interaction (*LRT, χ*^*2*^_*1*_ = *0.02, p* = *0.886*).

As no significant population-level biases were revealed, we analysed whether some individuals presented a significant individual-level bias. The binomial tests revealed that among the 11 individuals of the *Blue* + group, 2 showed a significant bias (*ID15* to the right*, p* < *0.001; ID17 *to the left*, p* = *0.016*) when faced with the blue colour choice, while other 2 individuals demonstrated a significant bias (*ID3* to the left, *p* = *0.005; ID9 *to the right*, p* = *0.027*) when faced with the yellow colour choice (Table 1). Among the 10 individuals in the *Yellow* + group, 1 demonstrated a significant bias (*ID10* to the right, *p* = *0.018*) when faced with the blue colour choice, while 4 individuals demonstrated a significant bias (*ID2* to the left, *p* = *0.016*; *ID8* to the left, *p* = *0.003*; *ID12* to the right, *p* = *0.016*; *ID16* to the right, *p* = *0.016*) when faced with the yellow choice (Table 1).

**Table 1:** Binomial test results showing a significant individual-level bias depending on which target colour was reward.

ID	Left Blue	Right Blue	*p* Blue	Left Yellow	Right Yellow	*p* Yellow	Number of choices	Rewarded target
1	7	4	0.161	8	4	0.121	23	
2	6	4	0.205	10	2	0.016*	22	
3	9	3	0.054	10	1	0.005**	23	
4	3	8	0.081	6	6	0.226	23	
5	4	8	0.121	3	9	0.054	24	
6	1	3	0.25	6	6	0.226	16	
7	4	8	0.121	6	6	0.226	24	
8	8	3	0.08	11	1	0.003**	23	
9	8	4	0.121	2	9	0.027*	23	
10	1	8	0.018*	9	3	0.054	21	
11	5	7	0.193	5	5	0.246	22	
12	2	4	0.234	2	10	0.016*	18	
13	8	4	0.121	6	5	0.226	23	
14	4	3	0.273	7	5	0.193	19	
15	0	12	< 0.001***	6	6	0.226	24	
16	6	4	0.205	2	10	0.016*	22	
17	10	2	0.016*	5	7	0.193	24	
18	6	3	0.164	9	3	0.054	21	
19	4	8	0.121	4	8	0.121	24	
20	3	3	0.313	4	8	0.121	18	
21	8	4	0.121	7	4	0.161	23	

The asterisks indicate significant difference: * for *p* < *0.05*; ** for *p* < *0.01*; *** for *p* < *0.001.*

When we merged all the trials (regardless the colour presented), out of the 21 individuals, 4 show a significant bias to the right (ID5, *p* = *0.021*; ID12, *p* = *0.012*; ID15, *p* = *0.008*; ID19, *p* = *0.044*) and 4 to the left (ID2, *p* = *0.009*; ID3, *p* = *0.001*; ID8, *p* = *0.001*; ID18, *p* = *0.026*) (Table 2).

**Table 2:** Binomial test results after merging all trials, showing a significant individual-level bias.

ID	Left picks	Right picks	Number of choices	*p*
1	15	8	23	0.058
2	16	5	21	0.018*
3	19	4	23	0.001***
4	9	14	23	0.097
5	7	17	24	0.021*
6	7	9	16	0.175
7	10	14	24	0.117
8	19	4	23	0.001***
9	10	13	23	0.136
10	10	11	21	0.168
11	10	12	22	0.154
12	4	14	18	0.012*
13	14	9	23	0.097
14	11	8	19	0.144
15	6	18	24	0.008**
16	8	14	22	0.076
17	15	9	24	0.078
18	15	6	21	0.026*
19	8	16	24	0.044*
20	7	11	18	0.121
21	15	0	23	0.058

The asterisks indicate significant difference: * for *p* < *0.05*; ** for *p* < *0.01*; *** for *p* < *0.001.*

**Figure 1:**
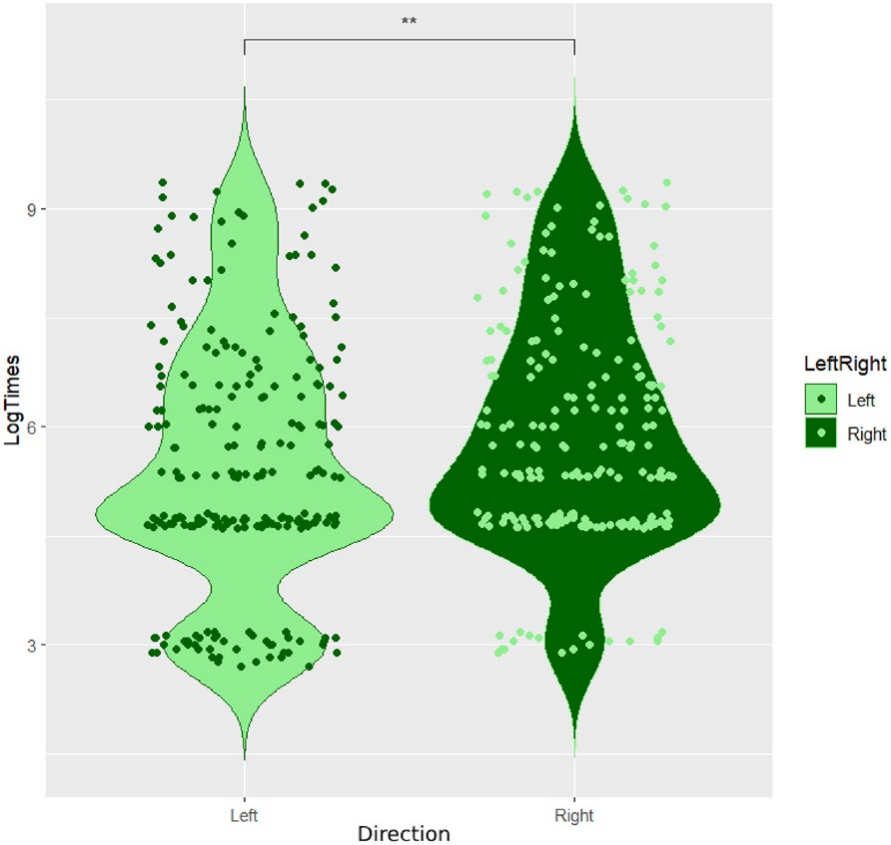
Violin plot of log-transformed times (in s) in the test choice of the left or right arm of the Y-maze (**p *< 0.01*).

**Figure 2:**
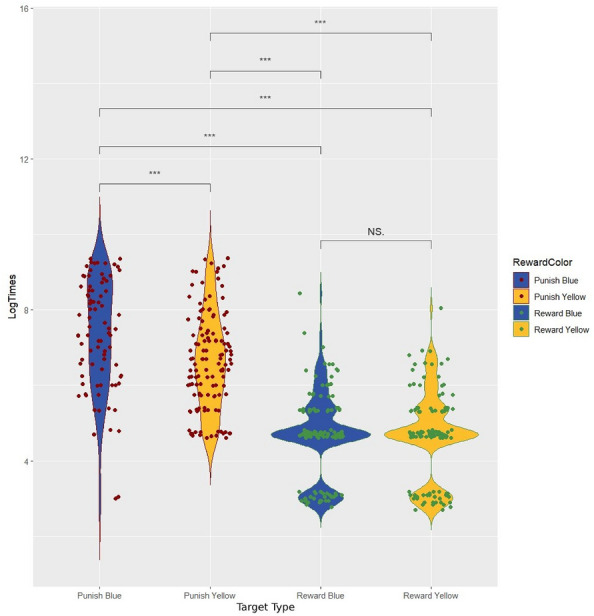
Violin plot of log-transformed times (in s) in the test choice of the blue or yellow target in relation to the association of these to the reward/punishment (***p < 0.001).

When the time taken to make a choice (i.e., to land on either the right or the left coloured stimuli) was considered, the data analysis found a significant effect of direction (*LRT, χ*^*2*^_*1*_ = *7.99, p* = *0.004; *Fig. 1)—with honeybees taking less time to reach the target in the left arm compared to the right arm—and a significant interaction between colour and reward/punishment (*LRT, χ*^*2*^_*1*_ = *5.34, p* = *0.02; *Fig. 2)—with honeybees taking less time to reach the rewarded targets compared to the punished ones and taking less time to reach the punished yellow target compared to the punished blue target –, but no significant 3-way interaction between colour, reward/punishment and direction (*LRT, χ*^*2*^_*1*_ = *1.33, p* = *0.249*), nor a significant interaction between direction and colour (*LRT, χ*^*2*^_*1*_ = *0.18, p* = *0.675*) or between direction and reward/punishment (*LRT, χ*^*2*^_*1*_ = *1.87, p* = *0.171*).

The average time taken by all bees to reach the targets on the right (*mean* ± *standard error: 12.88* ± *1.56 s; median* ± *IQR: 3.04* ± *9.02 s*) is greater than the average time taken to reach the targets on the left (*mean* ± *standard error: 10.67* ± *1.47 s; median* ± *IQR: 2.02* ± *6.17 s*). The effects of colour and reward/punishment are related, given the significance of the interaction. Post-hoc analysis revealed significance for the pairs*: Punish Yellow—Punish Blue (p* < *0.001); Reward Blue—Punish Blue (p* < *0.001); Reward Yellow—Punish Blue (p* < *0.001); Reward Blue—Punish Yellow (p* < *0.001); Reward Yellow—Punish Yellow (p* < *0.001),* but not *Reward Blue—Reward Yellow (p* = *0.999)* (Fig. 2). Regardless of the colour, the average time taken by all bees to reach the targets when punished (*mean* ± *standard error: 23.35* ± *2.07 s; median* ± *IQR: 10.05* ± *26.15 s*) is greater than the average time taken to reach the targets when rewarded (*mean* ± *standard error: 2.07* ± *0.25 s; median* ± *IQR: 1.1* ± *1.07 s*). In particular, the average time taken by *Yellow* + bees to reach the punished blue target (*mean* ± *standard error: 32.97* ± *3.75 s; median* ± *IQR: 16.14* ± *47.46 s*) is greater than the average time taken by *Blue* + bees to reach the punished yellow target (*mean* ± *standard error: 16.96* ± *2.21 s; median* ± *IQR: 7.2* ± *13.1 s*).

When investigating this colour difference by analysing the optical spectra of the two coloured stimuli, a difference in the reflectance was revealed (see Supplementary Material) with the yellow stimulus showing a greater reflectance for the UV component compared to the blue stimulus.

## Discussion

In this study we investigated lateralization in the directional choice of free-flying *Apis mellifera* in a foraging task: we did not study simple directional biases, but possible asymmetries linked to a visual-motor task, where the stimulus to be chosen had previously been associated with a sugar reward or a quinine punishment. As previous studies found behavioural asymmetries in honeybees and other related species, we expected the right arm to be overall preferentially chosen when the rewarded colour was presented and hypothesized that the punished colour would have been avoided when presented on the left, causing a potential rightward bias.

From our data instead emerged that the examined sample of 21 individuals did not show a directional bias at the population level, but only at the individual level, as some individuals (9 out of 21) had a significant rightward or leftward bias in stimulus choice. We instead found a population-level bias in flight speed (in terms of time spent on reaching the target) as overall honeybees took less time to choose the left arm than the right arm of the maze regardless the colour presented. Furthermore, as expected, bees were faster in choosing the colour previously associated with the sucrose reward than the colour previously associated with the quinine punishment (and this was true for both the *Blue* + and the *Yellow* + groups). What was unexpected was the effect of colour on the time honeybees took to choose the target previously associated with a punishment: punished yellow choices were overall faster than punished blue choices.

Our results are partially in line with previous findings in *Apis mellifera*^13,14^. In fact, Ong et al. already showed that honeybees present individual-level directional biases when they need to choose between a left and right aperture to fly through. Similarly, in our study only some individuals presented a significant bias to the right or to the left, whereas others did not show any bias. We need to consider that while Ong and colleagues investigated directional biases in a flying task, we presented the bees with a task where they had to choose the stimuli (in the right or the left arm of a maze) previously associated with a reward or a punishment and thus added a visual cognitive task to the simple motor task. A similar study in *Bombus terrestris,* where only one colour was associated with a reward and then presented in both maze’s arms^16^, suggests that bumblebees preferentially choose the right arm of the maze. We could not replicate the same result in honeybees likely because our experiment presented many more trials (12 for each colour) alternated with recall trials to keep the motivation high. If honeybees were trained only to associate one colour with a reward and presented only with that colour in subsequent unrewarded test trials (as done by Waite and Frasnelli^16^), it may be that they would show a similar rightward bias as bumblebees. Future experiments may uncover whether this is the case.

On the one hand, our results seem to support the idea that, in visuo-motor tasks, bees present individual biases (or absence thereof) which vary in strength and direction^14^, causing the population as a whole to not show any significant directional bias, because the polarities and strengths of individual bees’ biases cancel each other out^14^. We know that biases prove to be fundamental in various eco-ethological aspects: for example, if individuals in a population have different direction preferences for turning or passing through dense foliage, this can prevent "traffic jams" and slowdowns^14^; it can occur when it is necessary to enter unknown cavities^13^ or it is also useful in the speed with which a danger can be identified and faced. At the same time, an entire population sharing the same bias can be better synchronized and more efficient in daily activities: in bumblebees (*Bombus terrestris*) there is a strong right bias in foraging choices at the population level, suggesting a possible specialization of the right side of the nervous system for routine tasks^16^. However, it must be specified that the directional approaches of bees often depend on the context and the type of test to which they are subjected^6^. Indeed, when bumblebees are tested in spontaneous choices in a T-maze, they demonstrate no bias in turning direction^30^, although when within the same maze a test simulating their context of foraging is presented, the bias emerges, preferring to reach the target on their right rather than the one on their left^16^. On the other hand, the sensory modalities used by bees in crossing the labyrinths^13^ are lateralized to the right: the right antenna is richer in olfactory sensilla^23,31^ and is more efficient in odour learning and short-term memory recall^21,25,32^. Bees turn towards the odours of isoamyl-acetate, orange and lemon when presented to the right, while avoiding them (turning to the other side) when presented to the left^26^. Furthermore, bees are more sensitive to sugar when perceived with the right antenna than the left antenna^33^. This suggests that bees have more efficient biological characteristics to forage towards the right side, which reflects a visuo-motor bias in that direction^13,14^, but the study presented here did not find confirmations in this regard.

On the other hand, an unexpected result emerged from the analysis of the times employed in choosing the stimulus: honeybees took on average less time to choose the stimulus on the left than the one on the right, regardless of the colour presented and whether this had been previously associated with a reward or punishment. The speed bias towards the left could give new developments to the research on the laterality of *Apis mellifera*: the few previous studies^13,14,16^ on visual learning and motor orientation of this species have revealed a bias towards the right, but the present experiment leads us to consider a different perspective. It is true that bees show better foraging-related olfactory abilities through the right circuits, but in the light of the study presented, it would seem likely that the left side could be faster in the speed of choice, when the food source is known and does not require to be investigated further. A strong rightward bias has been documented in the exploration of unfamiliar cavities, where bees that turned right were faster in choosing than bees that went straight or turned left^13^. It must also be said that the task and the context, in this case, are different from the task of the study presented here and we know that the context is very important in the study of behavioural asymmetries^7^. If in the case of the rightward bias in the exploration of unknown cavities we talk about the exploration of a new environment where the right hemisphere (and the ipsilateral circuits connected to it) could guide the behaviour, in this study we consider a routine task where the left hemisphere could take over. Since the training phase was about 1–2 h long, and knowing that in bees, after 1 h, we can talk about long-term memory^24^, we can say that during all the testing trials bees used only long-term memory. Therefore, it is also worth highlighting that the effect of direction in speed (i.e., honeybees being faster in reaching the target on the left arm of the maze than on the right arm) may potentially be linked to the fact that long-term memory recall of the visual association is made through the left visuo-motor pathway as in the case of olfactory pathways^24^. In vertebrates it is well known that this cerebral specialization is conserved in the various species: the left hemisphere controls routine behaviours while the right hemisphere responds to new and unexpected stimuli^29^. Since in vertebrates the left hemisphere controls the right side of the body and vice versa (cross-lateral), while in invertebrates the left hemisphere controls the left side of the body (ipsi-lateral), these data would reveal an analogy between the two taxa: the left hemisphere would control routine tasks such as foraging in both taxa^28^. It should be noted, however, that the bias is only present in the speed of choosing and not in the number of choices, suggesting that the speed advantage to the left is not really exploited by making more choices to the left as in the case of exploration of unknown cavities, where right-turning bees (the majority) were also the quickest to choose^13^.

Furthermore, the data of this study revealed a strong effect of colour and its association with reward/punishment on choice times. We know that the blue colour is easier for bees to learn, having demonstrated an innate preference for stimuli with a green–blue wavelength^34,35^. Colours are essential signals in the choice of food foraged for the colony: both the ability of a single bee to recall a colour associated with positive reward^34^ and the ability to discriminate between colours^36,37^ are demonstrated. An interesting element which emerged from our research is the fact that the bees of the *Blue* + group had a smaller number of no-choices (namely 7, all corresponding to the punished yellow colour), compared to the bees of the *Yellow* + group (37 no-choices, all corresponding to the punished blue colour). This suggests that it is easier for honeybees not to choose blue, when associated with quinine, than yellow. Alternatively, it can be just easier to learn (or remember) associations with blue (punished or reinforced) than with yellow, but the results of the training did not reveal differences in the learning performance between the *Blue* + and the *Yellow* + groups. Another possible explanation for the difference in flight speed and no-choices number may relate to the different UV radiation of the two stimuli used in this study, as it could have influenced the decision (and flight) times of the tested subjects.

## Methods

### Subjects

This experiment was carried out between June 2022 and October 2022 at the SperimentArea complex, part of the Rovereto Civic Museum Foundation, whose spaces are granted for research purposes to CIMeC, University of Trento. SperimentArea hosts an apiary of four colonies of *Apis mellifera*. In this study, 21 foraging bees of *Apis mellifera* from these four hives were tested in free flight. To limit as much as possible the interaction with the experimenter—and, therefore, any stressful situations that could compromise the results of the tests—the bees were initially subjected to a group habituation process to the apparatus and then they were trained individually to enter the apparatus autonomously. Although we understand that bees belonging to the same colony may show a colony level lateralization (as suggested in ants), unfortunately we could not control for this variable. The hives were considered as one, because we worked with free-flight bees collected from a feeder and not from the hives, placed in a natural environment, with the aim of avoiding as much as possible manipulation by the experimenter and the possibility to induce stress.

### Experimental set-up

**Figure 3:**
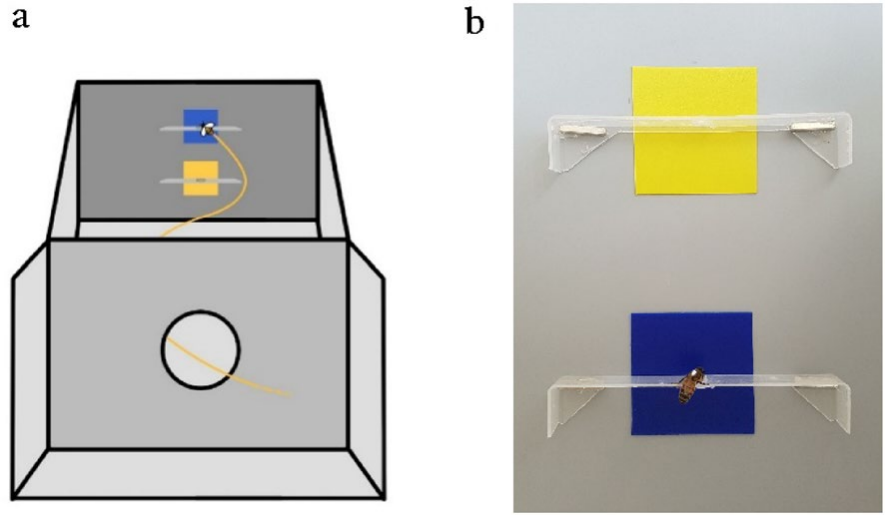
(**a**) The training apparatus with the coloured stimuli in above/below configuration (blue on top and yellow at the bottom in this case). The horizontal structures in front of the stimuli represent the landing platforms with a hole in the middle where the lids containing the sucrose, or the quinine solution, were fitted. (**b**) Training trial example: the white-marked bee is undergoing training to associate the blue stimulus with a reward and a punishment.

The experiments were run inside a wooden shelter (4 × 3 × 3 m, L × W × H) where we controlled for every possible environmental cue not to affect the directional choice made by the tested bees. The direction of the hives from the shelter was perfectly in line. The apparatus inside the shelter was perfectly centred to the entrance door as well. The main apparatus consisted of a wooden Y-maze, that could be dissembled in different modular chambers that were used for the training and the testing phases. The training apparatus allowed us to train the bees by placing the coloured stimuli in an above/below configuration (in order not to influence in any way the directionality investigated during the test phase), and it reproduced in shape and size the entrance to the Y-maze so that the bees could easily recognize the entrance of the Y-maze in the following testing phase (Fig. 3a).

**Figure 4:**
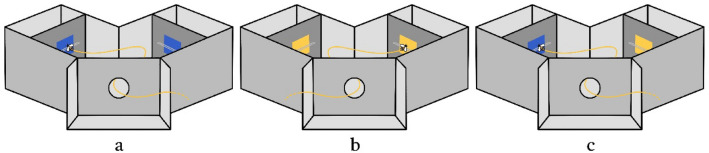
The Y-maze used in the testing phase: (**a**) blue–blue configuration, (**b**) yellow–yellow configuration; (**c**) blue–yellow configuration, used during the recall trails.

In detail, the training apparatus had an entrance consisting of a 50 mm hole (identical to the access hole to the Y-maze used in the testing phase) which could be closed with a sliding door between two aluminium guides, to prevent unwanted subjects from entering the apparatus. A 200 mm high mobile magnetic wall was used to place the stimuli in an above/below configuration (the "above" at 50 mm from the upper edge and the "below" at 50 mm from the lower edge). A removable and magnetic landing platform made of transparent plastic was used to dispense a sugar or quinine solution through a 4 mm hole in each stimulus’ centre, where the lid of a 0.2 ml Eppendorf tube was fitted (Fig. 3a). The test apparatus was composed by an entrance identical to the training apparatus and a Y-maze (Fig. 4). Two mobile magnetic walls equipped with a removable magnetic landing platform each, with the same characteristics as those used for the training, were placed in each arm of the maze. A recording camera (Sony DCR-SR57, 720p, f/1.8, 24fps) was placed on a tripod on top of the maze to film and time the testing phase.

The apparatus was mounted on a panel covered with white adhesive plastic foil, and the walls of the stimuli were covered with grey adhesive plastic foil, to allow cleaning the apparatus with a solution of 30% ethanol after every training and testing trial. Also, the landing platforms and the plastic-coloured stimuli were cleaned in the same way, thus ensuring hygiene and neutrality of odours inside the apparatus not to influence the choices of the bee through any previously released pheromones. The entire maze was covered by removable panels of 2 mm thick transparent polycarbonate, for optimal observation without any risk of bee escaping (or other bees entering).

The targets consisted of blue or yellow squared matte plastic tags measuring 50 mm × 50 mm (Fig. 3b). Depending on the individual tested, one colour was associated with a sugar solution, which acted as a positive reinforcement, as widely reported in the literature^38^, while the other colour was associated with a quinine solution, whose aversive function in bees (given by the very bitter taste) is widely recognized and, therefore, used as a negative stimulus^39,40^. Both percentages of the solutions used were selected based on previous studies^38–40^:

- 50% by weight for the sucrose solution, representing a high-value reward for bees;
- 2.5% by weight for the quinine solution, so that the strong bitter taste was perceptible, but remaining within the non-toxic limits for the species.

The study was run on two groups of honeybees: the *Blue* + group for which the blue colour was reinforced positively and the yellow was punished, and the *Yellow* + group for which the colour yellow was reinforced and the blue was punished.

The blue and yellow plastic tags were measured with a spectrophotometer (Avantes AvaSpec-2048, 386–928 nm) in the values of scope, absorbance and reflectance, using the AvaSoft 8 (version 8.16.1.0) software. All measurements have been done considering the possible light-filtering by the plexiglass cover.

### Experimental procedure

The bees familiarised with a feeder first and then each bee (one bee a day) was collected with a little plastic rod with a drop of sugar on top and carried to a landing pole filled with sugar solution and located in front of the circular entrance of the training module. By doing this, the bee was able to land independently on the landing pole. Subsequently, the landing pole was inserted inside the aforementioned module, so that it was visible from the entrance, in order to push the bee to enter the apparatus independently. After having entered and exited at least three times independently, the bee was placed on one of the transparent platforms (without any target) taking care to bring the landing pole close to the platform while the bee was still above it, intent on feeding. On the platform the bee still found sugar solution, so it learned that it was a safe place to land on. The positioning of the platforms was always alternated between "above" (i.e., 50 mm from the upper edge) and "below" (i.e., 50 mm from the lower edge) randomly. The centred above/below set up could avoid a left–right influence. Once the bee was well accustomed to the platforms, the blue and yellow targets were added, randomly arranged and the reinforcement or punishment solutions provided, depending on the group the tested individual belonged to: One colour was associated with the sugar solution, which acted as positive reinforcement, and the other with the quinine solution, which acted as punishment. The training procedure involved 10 trials in which the above/below arrangement of the targets was random in each trial and for each individual (Fig. 3a,b). The selected honeybee was marked with a dot of white UniPosca marker on the back and had to have a success rate of no less than 80% (8 out of 10 correct choices for the rewarded colour) in order to pass to the testing phase. For a success rate of less than 80%, the individual would have been discarded, but this never happened. Another important criterion to pass the training phase was for each bee to experience the quinine solution in order to learn the association of the colour with the punishment.

We collected 10 individuals for the *Yellow*+ and 11 for the *Blue*+*.*

The testing phase consisted of 24 testing trials alternated, two by two, with 12 recall trials (Fig. 4). The tests consisted in presenting two targets of the same colour, one on the wall placed in the right arm and one on the wall placed in the left arm of the Y maze, and record as choice the platform the bee touched first. In the test trials the platforms had only water, as a neutral stimulus. Two test trials were alternated with a recall trial, in which both colours (one on the right wall and the other on the left wall—counterbalanced for the relative position) were presented and either punished or rewarded. This was the optimal conformation to make sure that the motivation to return to the apparatus remained strong (if the bee always found only water, it would no longer have a reason to prefer the apparatus as a primary food source) and had the side effect to refresh the associations in the bee memory.

The value of 2 min and 0 s was set as the maximum time for each bee to make a choice, beyond which the apparatus was opened and the individual was free to exit, marking the attempt as "NA". Once the testing phase was completed, the individual was marked with a green colour not to re-subject it to the training in the following days.

Between the trials the bee returned to its hive and about 5–10 min later it came back autonomously to the apparatus. We looked at potential differences between *Blue*+ and *Yellow*+ between the test trials but, in both groups, bees showed no differences. Both the training and the testing trials did take only one day for a bee to finish all of them, so we could work with one different bee every day. On average it took each bee about one hour to familiarise with the apparatus and starting the training autonomously, about 2–3 h to complete the training (10 trials) and about 4–5 h to complete the testing phase (24 trials + 12 recalls).

### Data analyses

Adobe Premiere Pro software (release 2014) was used to process the videos obtained and the times for each individual test.

The analysis of the obtained data was conducted using the R software (version 4.3.0) with RStudio interface (version 2023.03.1). To evaluate differences in right-left directional choices in the testing phase, since this is a binary (categorical) variable, a binomial Generalized Linear Mixed-Effect Model was used with direction as the dependent variable, colour, reward/punishment and their interaction as effects and the ID of individuals as a random effect. For each individual tested and for each colour presented simultaneously in the right and left arm of the apparatus, a binomial test was then conducted using the online software *vassarstats.net* to evaluate lateralization at the individual level, i.e., if an individual made significantly more choices to the right or to the left for one colour or the other (or regardless the colour presented).

The times taken to complete the testing trials were analysed using a Generalized Linear Mixed-Effect Model with time as the dependent variable (log-transformed in order to ensure that the residuals were normally distributed), direction, colour, reward/punishment and their interactions as effects and the ID of individuals as a random effect.

In all statistical analyses, the value of *α* = *0.05* was used as the threshold of statistical significance.

### Supplementary Information

Supplementary Information 1.

Supplementary Information 2.

## Data Availability

All data generated and analysed during this study are included in this published article as a supplementary information file.
