# Supplementary material for: Visuo-motor lateralization in Apis mellifera: flight speed differences in foraging choices
Source: Sci Rep. 2024 Jan 5;14:660. doi: 10.1038/s41598-023-51141-w (PMC10770071; doi:10.1038/s41598-023-51141-w)
Supplement: Supplementary file 2 — Supplementary Information 2. [file 41598_2023_51141_MOESM2_ESM.pdf]

Visuo-motor lateralization in *Apis mellifera*: flight speed differences in foraging choices

Davide Liga [1], Gionata Stancher [2], Elisa Frasnelli [1,*]
[1] University of Trento, CIMeC, ING, Rovereto (TN), 38068, Italy
[2] Rovereto Civic Museum Foundation, Rovereto (TN), 38068, Italy
[*] elisa.frasnelli@unitn.it

SUPPLEMENTARY MATERIAL

We report here the values of scope, absorbance and reflectance of the blue and yellow plastic stimuli used during the experiment and measured with a spectrophotometer.

Scope:

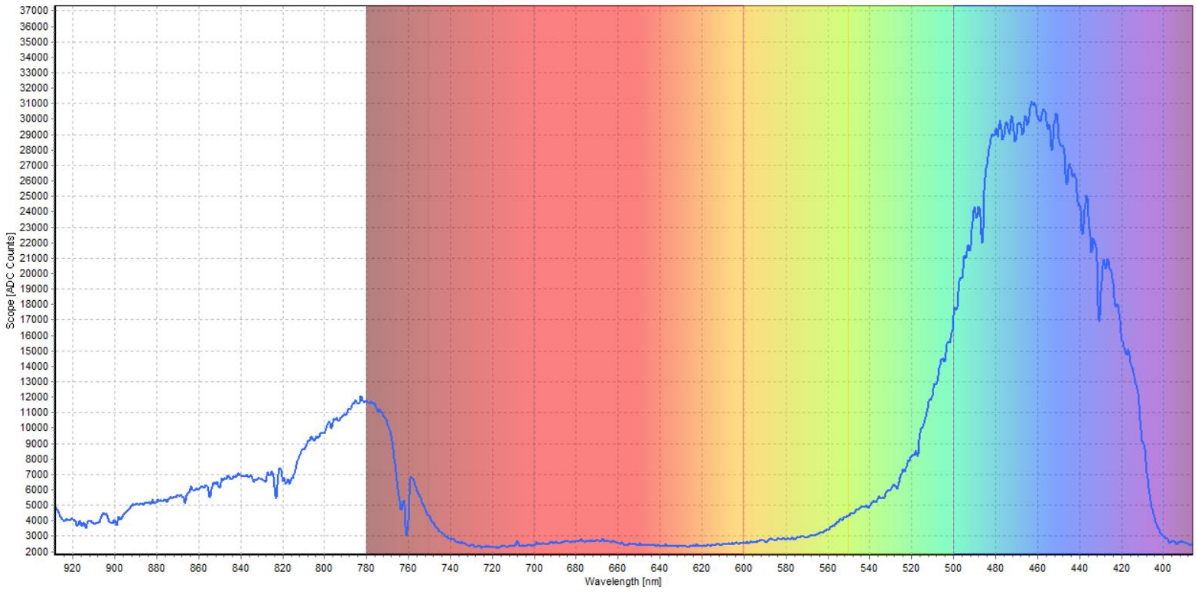

Figure S1. Blue target scope shows the peak at ~463 nm, the blue range of the visible spectrum.

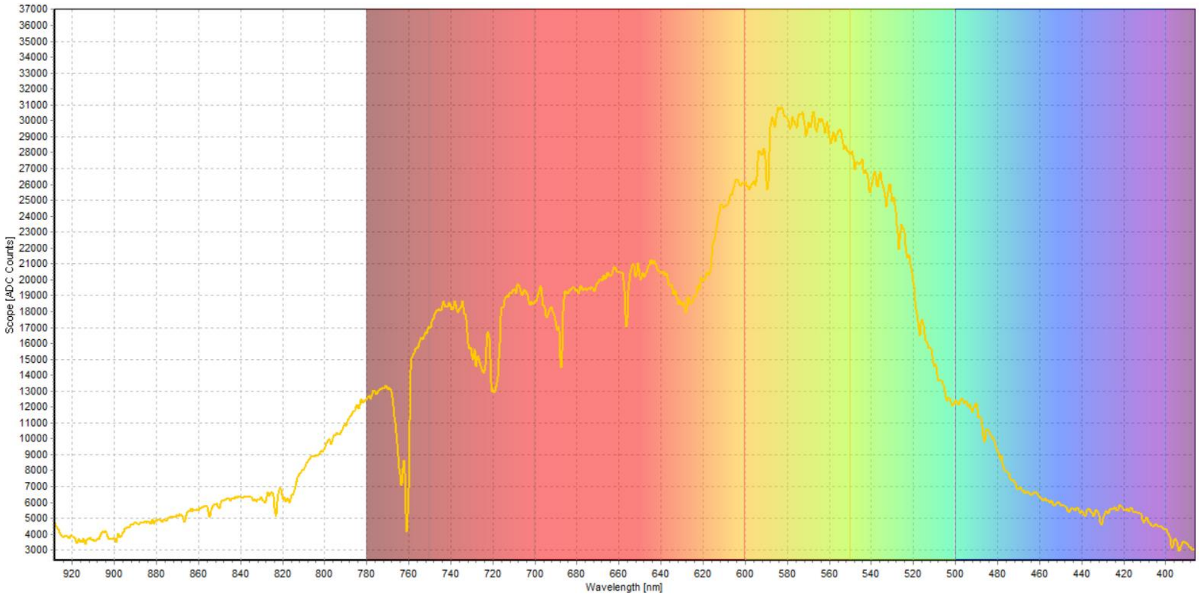

Figure S2. Yellow target scope shows the peak at ~584 nm, the yellow range of the visible spectrum.

Absorbance:

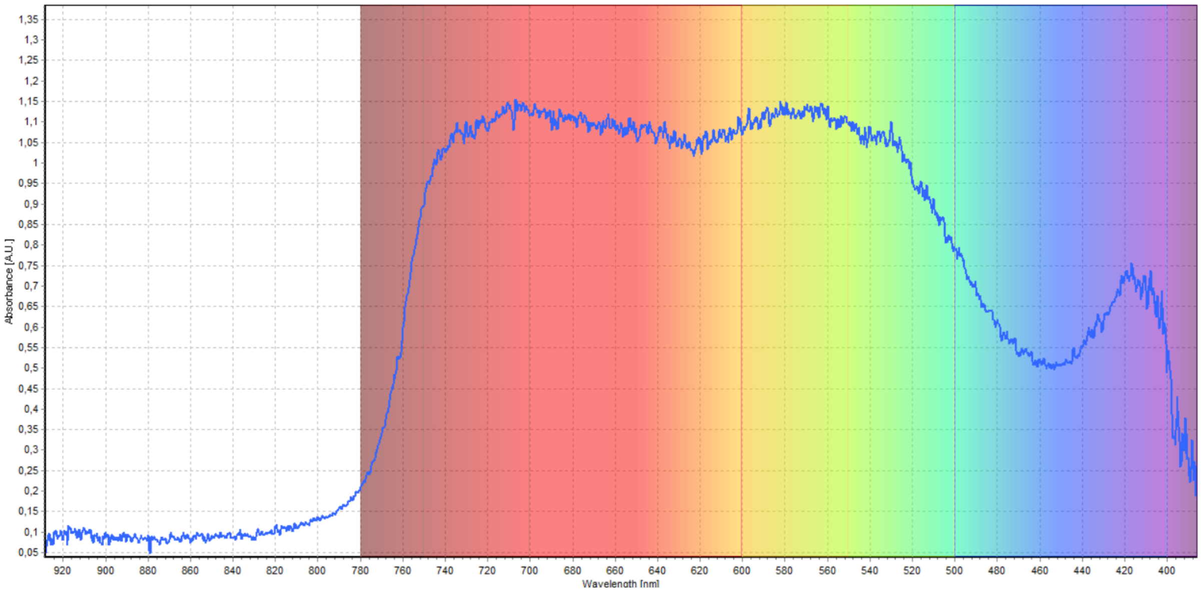

Figure S3. Blue target absorbance covers all the range from ~740nm to ~530nm of the visible spectrum, and also shows a new absorbance peak at ~418nm.

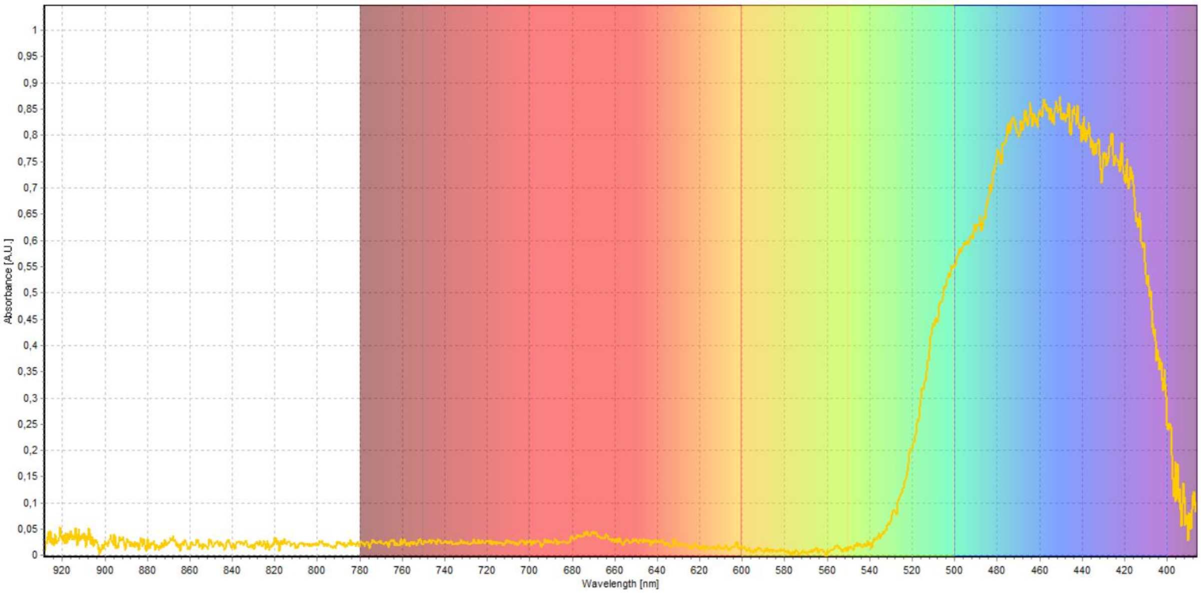

Figure S4. Yellow target absorbance covers the range from ~475nm to ~420nm of the visible spectrum.

<u>Reflectance:</u>

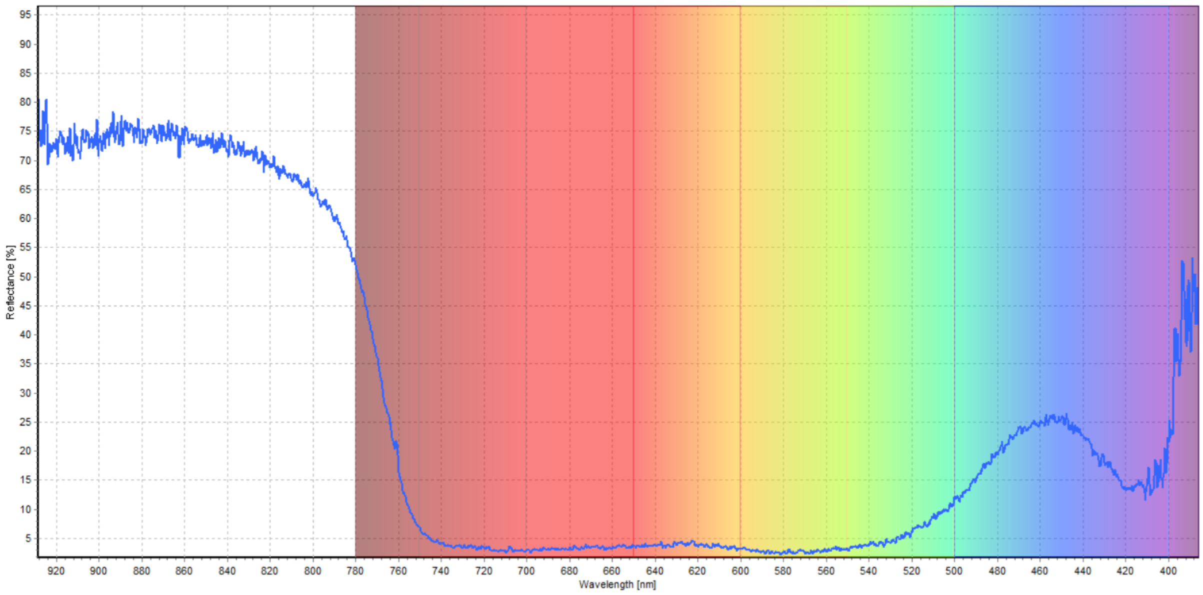

Figure S5. Blue target reflectance covers the range from ~460nm to ~450nm of the visible spectrum with a percentage of 25%, and also shows a 54% reflectance peak starting from ~390nm. The infrared peak is given by the hot temperature.

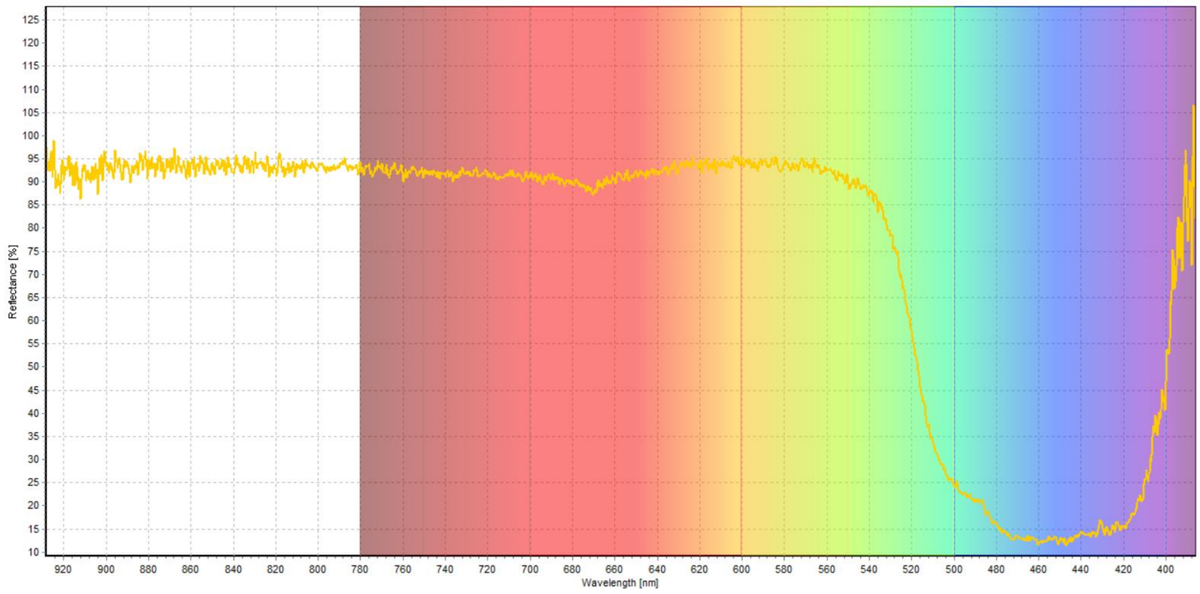

Figure S6. Yellow target reflectance covers all the range from ~780nm to ~540nm of the visible spectrum with a percentage of 94%, and also shows a 107% reflectance peak starting from ~390nm. The infrared peak is given by the hot temperature.
